# Supplementary material for: Thoracic UltrasONOgraphy Reporting: The TUONO Study
Source: J Clin Med. 2022 Nov 30;11(23):7126. doi: 10.3390/jcm11237126 (PMC9739733; doi:10.3390/jcm11237126)
Supplement: Supplementary file 1 [file jcm-11-07126-s001.zip › jcm-1963454-SI.pdf]

Supplementary materials
Panel S1: Items used to compare FTRs to SRs

ICU code: alphanumeric
Exam code: alphanumeric
Is it a free text report?: Y/N
Is it a standardized report?: Y/N
Is there a "notes section"? Y/N
Is patient diagnosis reported?: Y/N
Is the reason for examination described: Y/N
What is the reason for the examination? Free text or preselected items
Are images acquisition problems reported: Y/N
Is the type of ventilation (spontaneous breathing, support ventilation etc) reported? Y/N
Is the type of probe used reported? Y/N
Is patient position during examination reported? Y/N
Is the pleura investigated? : Y/N
Is the pleura described as normal? : Y/N
Is pleural aspect reported?
Is sliding reported?: Y/N
Is lung pulse reported?: Y/N
Is lung point reported?: Y/N
Are lung or chest zones clearly defined?: Y/N
Are basal zones the mainly or exclusively investigated zones?: Y/N
Is a vague localization of findings such as " on all the zones"- or "on the remaining zones" reported? Y/N
Is interception of anatomical lines (i.e., "on the third intercostal space on hemiclavear line"?) used to localize findings?: Y/N
Are anatomical terms (upper- lower lobe, apex/ base, etc.) used to localize findings? Y/N
Are findings wholly localized on the right or left or both hemithorax? Y/N
Is the number of defined zones on each hemithorax = 2 ? Y/N
Is the number of defined zones on each hemithorax = 3 ? Y/N
Is the number of defined zones on each hemithorax = 4 ? Y/N
Is the number of defined zones on each hemithorax = 6 ? Y/N
Are all the pre-established areas described? Y/N
Are single zones with no pathological sign described as normal ? Y/N
Are single zones with no pathological sign described as not investigated? Y/N
Is the exam mainly aimed to exclude a given pathology? Y/N
Does the exam exclude pleural or parenchymal alterations? Y/N
Does the exam exclude pleural alterations? Y/N
Does the exam exclude pneumothorax? Y/N
Does the exam exclude pleural effusion? Y/N
Does the exam exclude B-lines? Y/N
Is the exam aimed to exclude consolidations? Y/N

Does the exam exclude atelectasis? Y/N
Does the exam exclude air bronchogram? Y/N
Does the exam exclude static air bronchogram? Y/N
Does the exam exclude dynamic air bronchogram? Y/N
Does the exam exclude sub-pleural consolidation? Y/N
Is the finding described as “normal”? Y/N
Is the finding described as “A lines”? Y/N
Is the finding described as “B-lines”? Y/N
Is the finding described as “consolidation”? Y/N
Is the finding described as “static air bronchogram“? Y/N
Is the finding described as “dynamic air bronchogram “? Y/N
Is the finding described as “atelectasis“? Y/N
Is the finding described as “subpleural consolidation“? Y/N
Is the finding described as “pneumonia“? Y/N
Is the finding described as “contusion“? Y/N
Is the finding described as “PNX“? Y/N
Is the finding described as “effusion“? Y/N
Is the finding described as “curtain-sign“? Y/N
are other terms used instead of “B-lines” (i.e “lung rockets”, “pulmonary congestion” “fluid overload”)? Y/N
Are B lines reported without any other specification? Y/N
Is the number of B lines reported? Y/N
Are B-lines quantified as few or many?
Are B-lines described as defined or crowding?
Is effusion reported without any other specification? Y/N
Is effusion quantified as minimal, little, abundant, massive etc?
Is effusion extent measured (i.e cms) or described (i.e n° of intercostal spaces)? Y/N
Is the effusion volume (milliliters) estimated?
Is the effusion aspect described? Y/N
Is the effusion echogenicity described? Y/N
Is consolidation reported without any other specification? Y/N
Is consolidation quantified as minimal, small, large, etc.? Y/N
Are consolidation dimensions measured in centimeters? Y/N
Is bronchogram reported without any other specification? Y/N
Is bronchogram reported as static? Y/N
Is bronchogram reported as dynamic? Y/N
Is atelectasis reported without any other specification? Y/N
Is atelectasis quantified as minimal, small, large, etc.? Y/N
Is extravascular lung water estimated? Y/N
Which terms are used to estimate extravascular lung water?
Is there a modification of signs during breathing or ventilation? Y/N
Is diaphragm investigated? Y/N
Is diaphragm uplift reported? Y/N

Is diaphragm thickening or motility described in qualitative terms? Y/N
Is diaphragm thickening or motility measured in millimeters / centimeters? Y/N
Is Lung Ultrasound Score reported?
Is a conclusions section present? Y/N
Is there a comparison with previous exams? Y/N
Are there any treatment changes generated by ultrasound examination described? Y/N
Which treatment changes have been generated by the exam?
Are there other diagnostic tests to be performed in the report?
Are the results of an echocardiography also shown in the report? Y/N
Is there an inferior vena cava evaluation? Y/N
Is there a diagnostic hypothesis? Y/N

Table S1. Differences in SR and FTR characteristics.

	Tot (n - %)	SRs (n - %)	FTRs (n - %)	Fisher exact test (p)
ICU admission diagnosis	47 (27%)	34 (58%)	13 (12%)	< 0.001
Reason for examination	44 (26%)	40 (68%)	4 (4%)	< 0.001
Quality of US window	17 (10%)	2 (3%)	15 (13%)	0.06
Type of mechanical ventilation	71 (42%)	36 (61%)	35 (31%)	< 0.001
Type of probe	53 (31%)	40 (68%)	13 (12%)	< 0.001
Patient position	71 (42%)	26 (44%)	45 (40%)	0.6

Table S2. Conditions ruled-out in targeted examinations.

Conditions to be ruled out	Tot N = 19	SRs N = 2	FTRs N = 17
Pleuro-parenchymal lesions	10/19	0	10/17
Pleural alterations	1/19	0	1/17
Pneumothorax	4/19	2/2	2/17
Pleural effusion	15/19	2/2	13/17
B-lines	5/19	1/2	4/17
Consolidations	4/19	2/2	2/17
Atelectasis	2/19	1/2	1/17

Table S3. Diaphragm features.

Item	Tot	SRs	FTRs	Fisher exact test (p)
Diaphragm	29/171 (17%)	11/59 (19%)	18/112 (16%)	0.6733
Diaphragm uplift	7/29 (24%)	1/11 (9%)	6/18 (33%)	0.1912
Diaphragm Motility or thickening	22/29 (76%)	10/11 (91%)	12/18 (67%)	0.2021
Diaphragm Thickening (mm/cm)	0	0	0	-

Table S4. Other features.

Item	Tot (n=171)	SRs n (n=59)	FTRs (n=112)	Fisher exact test (p)
Echocardiography	33/171 (19%)	15/59 (25%)	18/112 (16%)	0.16
Inferior vena cava analysis	29/171 (17%)	10/59 (17%)	19/112 (17%)	1.00
Pulmonary water content estimation	20/171 (12%)	9/59 (15%)	11/112 (10%)	0.32
